# Investigating and Improving the Accuracy of US Citizens’ Beliefs About the COVID-19 Pandemic: Longitudinal Survey Study

**DOI:** 10.2196/24069

**Published:** 2021-01-12

**Authors:** Aart van Stekelenburg, Gabi Schaap, Harm Veling, Moniek Buijzen

**Affiliations:** 1 Behavioural Science Institute Radboud University Nijmegen Netherlands; 2 Erasmus School of Social and Behavioural Sciences Erasmus University Rotterdam Rotterdam Netherlands

**Keywords:** infodemic, infodemiology, misinformation, COVID-19 pandemic, belief accuracy, boosting, trust in scientists, political orientation, media use

## Abstract

**Background:**

The COVID-19 infodemic, a surge of information and misinformation, has sparked worry about the public’s perception of the coronavirus pandemic. Excessive information and misinformation can lead to belief in false information as well as reduce the accurate interpretation of true information. Such incorrect beliefs about the COVID-19 pandemic might lead to behavior that puts people at risk of both contracting and spreading the virus.

**Objective:**

The objective of this study was two-fold. First, we attempted to gain insight into public beliefs about the novel coronavirus and COVID-19 in one of the worst hit countries: the United States. Second, we aimed to test whether a short intervention could improve people’s belief accuracy by empowering them to consider scientific consensus when evaluating claims related to the pandemic.

**Methods:**

We conducted a 4-week longitudinal study among US citizens, starting on April 27, 2020, just after daily COVID-19 deaths in the United States had peaked. Each week, we measured participants’ belief accuracy related to the coronavirus and COVID-19 by asking them to indicate to what extent they believed a number of true and false statements (split 50/50). Furthermore, each new survey wave included both the original statements and four new statements: two false and two true statements. Half of the participants were exposed to an intervention aimed at increasing belief accuracy. The intervention consisted of a short infographic that set out three steps to verify information by searching for and verifying a scientific consensus.

**Results:**

A total of 1202 US citizens, balanced regarding age, gender, and ethnicity to approximate the US general public, completed the baseline (T0) wave survey. Retention rate for the follow-up waves— first follow-up wave (T1), second follow-up wave (T2), and final wave (T3)—was high (≥85%). Mean scores of belief accuracy were high for all waves, with scores reflecting low belief in false statements and high belief in true statements; the belief accuracy scale ranged from –1, indicating completely inaccurate beliefs, to 1, indicating completely accurate beliefs (T0 mean 0.75, T1 mean 0.78, T2 mean 0.77, and T3 mean 0.75). Accurate beliefs were correlated with self-reported behavior aimed at preventing the coronavirus from spreading (eg, social distancing) (*r* at all waves was between 0.26 and 0.29 and all *P* values were less than .001) and were associated with trust in scientists (ie, higher trust was associated with more accurate beliefs), political orientation (ie, liberal, Democratic participants held more accurate beliefs than conservative, Republican participants), and the primary news source (ie, participants reporting CNN or Fox News as the main news source held less accurate beliefs than others). The intervention did not significantly improve belief accuracy.

**Conclusions:**

The supposed infodemic was not reflected in US citizens’ beliefs about the COVID-19 pandemic. Most people were quite able to figure out the facts in these relatively early days of the crisis, calling into question the prevalence of misinformation and the public’s susceptibility to misinformation.

## Introduction

Public health crises tend to go hand in hand with information crises. The COVID-19 pandemic, which is taking many lives and is hospitalizing hundreds of thousands of people globally, is no exception. In the wake of the COVID-19 pandemic, we are seeing signs of a misinformation pandemic. Around the first peak of the coronavirus outbreak in the United States, the country with the highest COVID-19 death toll [1], about two-thirds of Americans said they had been exposed to at least some made-up news and information related to the virus [2]. Misinformation about the pandemic seems to have proliferated quickly, especially on social media [3]. The World Health Organization (WHO) has labelled this surge of information and misinformation about the COVID-19 pandemic an *infodemic* [4].

Countries and social media platforms are trying to tackle this infodemic in a number of ways. Several social media platforms, including Facebook and Twitter, have implemented new procedures to remove or label false and misleading content [5,6]. However, with the vast number of posts made to these platforms every day and the platforms’ fear of infringing on free speech, the success of these procedures is limited (eg, [7]). A second strategy consists of surfacing trusted content, for instance, by referring people with questions to the WHO or to national health agencies, such as the Centers for Disease Control and Prevention in the United States and the National Epidemiology Center in Brazil. This approach might be hindered by government officials, including US president Donald Trump and Brazilian president Jair Bolsonaro, actually contributing to the spread of misinformation (eg, [8,9]). Considering this apparent infodemic, are people able to distinguish facts from fiction? And what correlates might enable or disable them in forming accurate beliefs?

One promising approach to limiting the effects of misinformation was already on the rise before the COVID-19 pandemic: increasing misinformation resistance through educational interventions. A substantial number of countries have implemented educational interventions, primarily focused on *media literacy* [10], which can be understood as the ability to access, analyze, evaluate, and communicate messages in a variety of forms [11]. The Swedish Civil Contingencies Agency, for instance, has included a section about misinformation in its public emergency preparedness brochure, advising Swedes to be aware of the aim of information and to check the source of information, among others [12]. Similarly, Facebook tries to help its users recognize misinformation by providing 10 tips [13]. One advantage of such a focus on media literacy is that it can help prevent problems with misinformation, instead of having to correct false beliefs after they have taken hold. Previous media literacy research, with interventions focusing on identification of misinformation, has yielded promising results indicating that some interventions can reduce the perceived accuracy of misinformation [14,15]. Other research highlights the difficulties in crafting media literacy interventions [16]. Can these types of interventions, focusing on empowerment of media consumers, help individuals deal with the supposed COVID-19 infodemic?

Our approach focuses on helping individuals figure out what is true and what is false, considering false such beliefs about factual matters that are not supported by clear evidence and expert opinion [17]. We test an intervention that empowers people to search for and identify scientific consensus. Communicating scientific consensus (ie, a high degree of agreement between scientists) is effective in eliciting scientifically accurate beliefs [18]. This effectiveness is described in the gateway belief model, which states that people’s perceived scientific consensus functions as a gateway to their personal factual beliefs [19,20]. Here, we focus on empowering individuals to search for and identify scientific consensus, because this approach is more flexible than communicating a scientific consensus on every single issue.

The current strategy is considered a *boosting* approach. Boosting encompasses interventions targeting competence rather than immediate behavior [21]. In line with this, our intervention focuses on improving people’s skills to form accurate beliefs, instead of altering the external context within which people form beliefs. In addition, our boosting approach can be considered an educational intervention, just like media literacy interventions. However, compared to media literacy interventions that target the identification of misinformation, boosting *consensus reasoning* is not dependent on being exposed to misinformation. One can investigate any claim, true or false, from any source.

This study had two main goals. One involved an exploratory, not preregistered, investigation to gain insight into the effects of the supposed infodemic on individuals’ belief accuracy in times of crisis, and to investigate potential correlates of belief accuracy. The second goal was a preregistered test of the boosting intervention aimed at increasing belief accuracy. Accordingly, we hypothesized that our intervention would lead to more accurate beliefs about the COVID-19 pandemic than the control condition; the complete preregistration can be found on the Open Science Framework (OSF) [22]. The research was conducted online, and a balanced sample of the US population was recruited. We decided to focus on the United States, because this is arguably the country worst hit by the COVID-19 pandemic. Using a longitudinal design, measuring beliefs about the pandemic over 4 weeks just after daily confirmed COVID-19 deaths had peaked at over 4000, allowed us to investigate belief formation in the relatively early days of the pandemic. All data and material are available on the project page on the OSF [23].

## Methods

### Recruitment

We used Prolific, a UK-based online crowdsourcing platform that connects researchers to participants, to collect data from US citizens over a 4-week period. Prolific has been demonstrated to yield high-quality data and more diverse participants than student samples or other major crowdsourcing platforms [24]. In addition, it allowed for recruitment that balanced age, gender, and ethnicity to approximate the US general public, via stratification using US census data [25]. Recruitment for the initial baseline wave started on April 27, 2020.

A total of 1212 individuals participated in the study at baseline (T0), for which they received £0.45 (~US $0.56). A total of 1089 individuals participated in the first follow-up wave (T1), 1070 individuals participated in the second follow-up wave (T2), and 1028 individuals participated in the final wave (T3); see Table 1 for total sample size, exclusions, and final sample size per wave. Participants received £0.33 (~US $0.41) for participation per follow-up survey. Each of the waves was separated by approximately one week (mean_T0-T1_ 6.98 days, SD _T0-T1_ 14.92 hours; mean_T1-T2_ 7.01 days, SD _T1-T2_ 12.72 hours; mean_T2-T3_ 7.06 days, SD _T2-T3_ 13.18 hours). The sample size was determined by the available resources. This study is part of a research project that was reviewed and approved by the Ethics Committee Social Science at Radboud University (reference No. ECSW-2018-056).

**Table 1:** Total sample size, exclusions, and final sample size per wave.

Wave and sample		Value, n (%)	Retention rate, %^a^
**T0 (baseline): April 27-29, 2020**			
	Total sample	1212 (100)	N/A^b^
	Excluded^c^	10 (0.8)	N/A
	Final sample	1202 (99.2)	N/A
**T1 (first follow-up wave): May 4-7, 2020**			
	Total sample	1089 (100)	N/A
	Excluded^c^	11 (1.0)	N/A
	Final sample	1078 (99.0)	89.7
**T2 (second follow-up wave): May 11-14, 2020**			
	Total sample	1070 (100)	N/A
	Excluded^c^	3 (0.3)	N/A
	Final sample	1067 (99.7)	88.8
**T3 (final wave): May 18-21, 2020**			
	Total sample	1028 (100)	N/A
	Excluded^c^	6 (0.6)	N/A
	Final sample	1022 (99.4)	85.0

^a^The retention rate is based on the final sample sizes of T0 and the respective wave. Total sample sizes of follow-up waves were counted, excluding 2 participants who should have been excluded but had been allowed to participate in the follow-up waves due to a technical error.

^b^N/A: not applicable; retention rates were calculated using final sample sizes of T0 and the follow-up waves.

^c^More details on exclusions can be found in the Statistical Analysis, Data Exclusion section.

### Procedure

**Figure 1:**
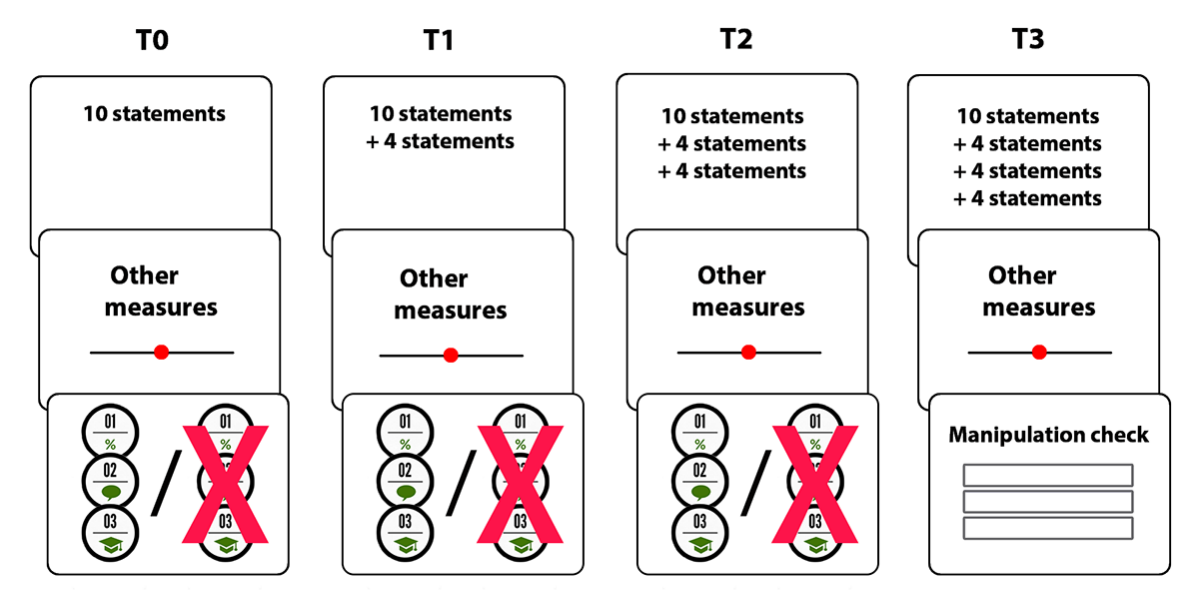
Flowchart of the main elements of the procedure per wave. Participants first completed the measure of belief accuracy, then completed other measures, and, finally, were exposed to the intervention or not. At T3, participants completed a manipulation check. The bottom panels of the first three columns display the intervention condition (left; infographic) and the control condition (right; no intervention). T0: baseline; T1: first follow-up wave; T2: second follow-up wave; T3: final wave.

Participants were randomly assigned to either receive the intervention (ie, the boost condition) or no intervention (ie, the control condition). All surveys started with the measure of belief accuracy, for which participants were presented with 10 (T0), 14 (T1), 18 (T2), or 22 (T3) statements about the coronavirus and COVID-19 (see Figure 1). Participants indicated to what extent they believed each statement to be true. An attention check was included among these statements (see Multimedia Appendix 1). Subsequently, participants reported their behavior aimed at preventing the spread of the coronavirus and completed the other measures. The boosting intervention, an infographic presenting three steps that can be used to evaluate a claim, was included at the end of T0, T1, and T2. Only participants in the boost condition were presented with the infographic, allowing them to apply their boosted consensus reasoning skill in the week leading up to the next wave. At T3, all participants completed a manipulation check. At the end of T0, all participants entered demographic information and completed a seriousness check (see Multimedia Appendix 1). All surveys took about 3 to 6 minutes.

### Materials and Measures

#### Belief Accuracy

The key dependent variable was the accuracy of participants’ beliefs related to the COVID-19 pandemic. This variable consisted of responses to a number of statements about the pandemic, which were sourced from preprints of early research on public perceptions of COVID-19 (eg, [26]), public health agencies and medical institutes (eg, the WHO), media tracking organizations (eg, NewsGuard), and expert reports in established media (eg, CNBC); a comprehensive list of these resources is available in Multimedia Appendix 2. Only statements based on scientific claims were included in order to make sure that there was compelling evidence that the claims were either true or false.

At T0, participants were exposed to 10 statements, of which five were scientifically accurate (eg, “Fever is one of the symptoms of COVID-19”) and five were at odds with the best available evidence (eg, “Radiation from 5G cell towers is helping spread the coronavirus”). Participants responded by indicating the accuracy of a statement as follows: *false*, *probably false*, *don’t know*, *probably true*, or *true*. In each subsequent wave, four new statements were added to the list of statements: two accurate ones and two inaccurate ones. This allowed us to keep the belief accuracy measure current, reflecting contemporary insights and discussion points. The order of the statements was randomized per participant and varied per wave.

A belief accuracy score was calculated by converting the response to each statement to a number reflecting how accurate the response was; a correct judgment was counted as 1 and an incorrect judgment was counted as –1. A less certain but correct *probably true* or *probably false* counted as 0.5 and an incorrect one as –0.5. Finally, a *don’t know* response was counted as 0. Average scores were calculated per wave per participant, resulting in a repeated measure of belief accuracy. Internal consistency was acceptable to good across the four waves; the McDonald ω_t_ was between 0.75 and 0.87 in all waves.

#### Coronavirus-Related Behavior

Coronavirus-related behavior aimed at preventing the coronavirus from spreading was measured by asking participants to indicate their agreement with three statements. The statements were “To prevent the coronavirus from spreading...” (1) “I wash my hands frequently,” (2) “I try to stay at home/limit the times I go out,” and (3) “I practice social distancing (also referred to as ‘physical distancing’) in case I go out”; agreement was measured on a scale from 1 (strongly disagree) to 7 (strongly agree). Scores were averaged per wave per participant. Internal consistency was acceptable to good across the four waves; the McDonald ω_t_ was between 0.77 and 0.83 in all waves.

#### Additional Measures

Trust in scientists was measured in all four waves with responses to the statement “I trust scientists as a source of information about the coronavirus.” Participants responded on a 7-point scale ranging from 1 (strongly disagree) to 7 (strongly agree).

Participants’ primary news source for information about the COVID-19 pandemic was identified by asking them at T0 what their main source of news about the coronavirus was. Participants could choose one option from a list of 11 news sources, based on data from the Pew Research Center on Americans’ news habits [27].

Finally, we included a manipulation check at T3. This consisted of asking participants how they evaluated the truthfulness of the statements about the coronavirus and coronavirus disease in the study over the past weeks. We asked them to name the steps that they took to evaluate the claims in three open text boxes, of which at least one had to be used. These answers were coded by the first author to indicate whether they mention consensus—or something similar—or not. A second coder coded a random subset of 120 answers, with Krippendorff α indicating good (α=.85) interrater reliability. Therefore, the complete coding from the first author was used in the analyses.

Not all measures included in the study are listed here, because not all measures are relevant here. Please see the material on the project page on the OSF for the remaining measures [23].

#### Intervention

The boosting intervention that was included at the end of T0, T1, and T2 consisted of a short infographic that was aimed at empowering participants to use scientific consensus when evaluating claims related to the COVID-19 pandemic. The infographic set out three steps that can be used to evaluate a claim: (1) searching for a statement indicating consensus among scientists, (2) checking the source of this consensus statement, and (3) evaluating the expertise of the consensus. The infographic can be found in Multimedia Appendix 3. Participants in the control condition were not exposed to the infographic.

#### Demographics

Demographics including political orientation, age, gender, ethnicity, and education were asked about at T0. Political orientation was measured by combining political identity (ie, strong Democrat, Democrat, independent lean Democrat, independent, independent lean Republican, Republican, or strong Republican) and political ideology (ie, very liberal, liberal, moderate, conservative, or very conservative) into one numeric, standardized measure centered on 0 (ie, moderate, independent), based on Kahan [28].

### Statistical Analysis

#### Data Exclusion

First, we removed one of two duplicate responses at T1 and excluded all responses from one participant with three varying responses at T3.

As preregistered, participants who failed the attention check at T0 were excluded and replaced (n*=*8; including 2 who had been allowed to participate in the follow-up waves due to a technical error). If a participant failed one of the attention checks in the subsequent waves, data from that wave was not included in the analyses (n_T1_*=*5, n_T2_*=*2, n_T3_*=*5), but other surveys in which the attention check was passed were retained. Participants who indicated at the T0 seriousness check that their data should not be used were excluded from further participation and their data were not used, but they were not replaced (n=2). No participants completed T0 in less than 1 minute, but if a participant completed a subsequent wave in less than 1 minute, data from that survey were not included in the analyses (n_T1_=6, n_T2_=1, n_T3_=0). Other waves in which the 1-minute threshold was passed were retained.

#### Exploratory Analyses

General increase in belief accuracy over time was explored using linear mixed modeling for each set of statements, with wave as predictor, controlling for political orientation and including a random intercept per participant. The relationship between belief accuracy and coronavirus-related behavior was explored with correlations for each wave. The relationship of belief accuracy with trust in scientists at T0, political orientation, and primary news source was explored using mixed modeling, controlling for wave, age, gender, education, and ethnicity. The interaction term between trust and political orientation was included in the model. The five most chosen news sources (ie, CNN, Fox News, NPR, social media sites, and The New York Times, excluding the option *Other sources*) were included as dummy-coded variables. Finally, we included a random intercept and a random slope for wave per participant. Mixed modeling was performed with the lme4 package [29] in R (The R Foundation) [30]. The models were examined using likelihood ratio tests, using the R package lmerTest [31].

#### Preregistered Analysis

The hypothesis that our intervention would lead to more accurate beliefs than would the control condition was also tested using linear mixed modeling. The condition (ie, intervention vs control) and wave, and the interaction between condition and wave, were included as predictors in the model. Political orientation was included as a covariate, because beliefs about the COVID-19 pandemic are related to political ideology [32], and a random intercept and a random slope for wave were included per participant. The hypothesis was tested by comparing the full model, with the interaction between condition and wave, to a model without this interaction effect. We used the PBmodcomp function from the R package pbkrtest [33] for parametric bootstrapping (10,000 simulations).

## Results

### Participants

The final sample roughly reflects US census data [25] on gender, age, and ethnicity, indicating that the balanced sampling worked well. See Table 2 for more details.

**Table 2:** Participant characteristics.

Demographic characteristic			Sample value (N=1202), n (%)^a^		Census value, %^b^
**Gender**					
	Female	604 (50.2)		51.3	
	Male	587 (48.8)		48.7	
	Other	11 (0.9)		N/A^c^	
**Age in years**					
	18-24	164 (13.6)		11.9	
	25-34	243 (20.2)		17.9	
	35-44	209 (17.4)		16.4	
	45-54	199 (16.6)		16.0	
	55-64	232 (19.3)		16.6	
	65-74	139 (11.6)		12.4	
	≥75	16 (1.3)		8.8	
**Ethnicity**					
	White	918 (76.4)		73.6	
	Black	158 (13.1)		12.5	
	Asian	79 (6.6)		5.9	
	Mixed	30 (2.5)		2.5	
	Other	17 (1.4)		5.5	

^a^Due to rounding, percentages may not add up to 100% exactly.

^b^The percentages in the census data reflect the population aged 18 years and over.

^c^N/A: not applicable.

### Belief Accuracy

Mean scores of belief accuracy were very high for all waves, with scores reflecting low belief in false statements and high belief in true statements. There was substantial variation in the accuracy of responses between statements, although none of the statements was ever interpreted with less than 0.25 accuracy, on average; see Multimedia Appendix 4 for a complete overview of scores per statement per wave.

**Figure 2:**
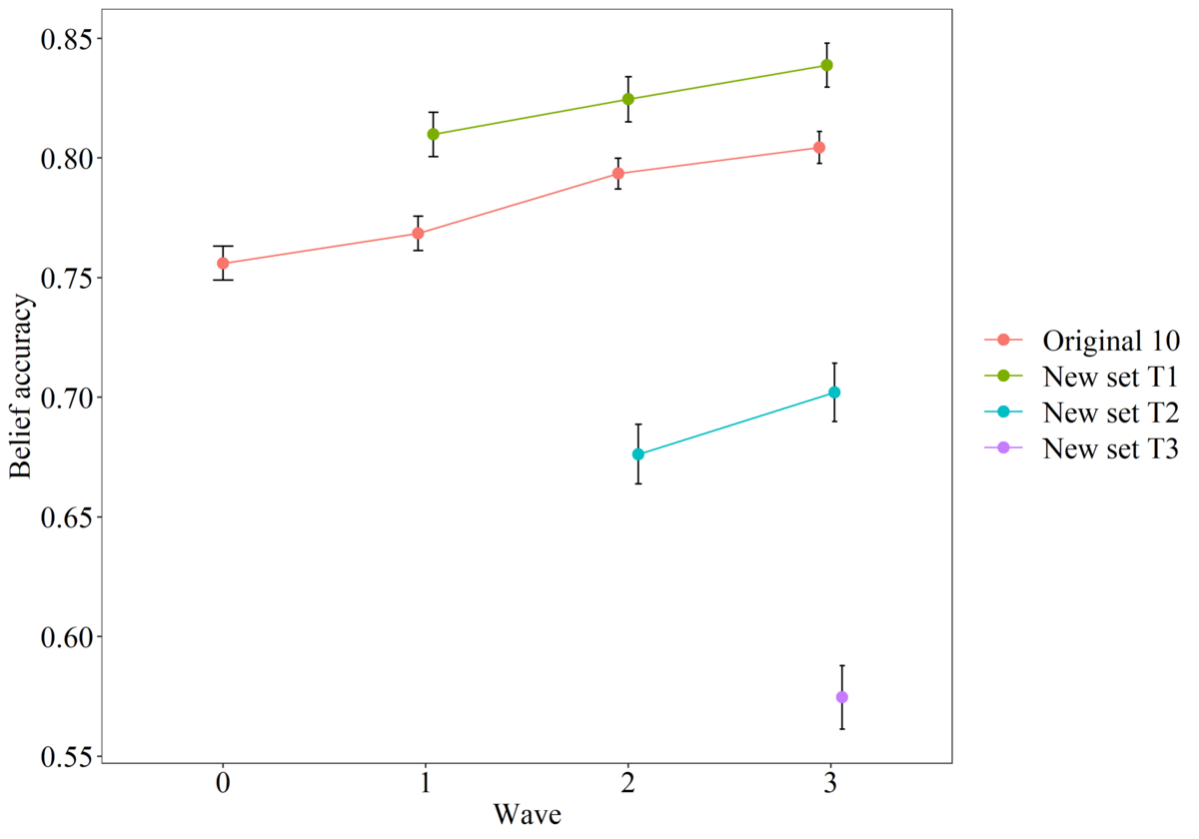
Belief accuracy per set of statements over time. The new set at T3 was included for completeness. Focusing on within-subject change, dots represent normed means and error bars indicate 95% CIs of the within-subject SE [34], calculated using the summarySEwithin function from the Rmisc package [35]. The belief accuracy scale ranged from –1, indicating completely inaccurate beliefs, to 1, indicating completely accurate beliefs. T1: first follow-up wave; T2: second follow-up wave; T3: final wave.

There was a modest increase in belief accuracy over time, looking at each set of statements separately (first 10: estimate=0.02, SE<0.01; t_3202.59_=13.82, *P*<.001; T1 set: estimate=0.01, SE<0.01; t_2041.94_=4.80, *P*<.001; T2 set: estimate=0.02, SE<0.01; t_1003.22_=3.40, *P*<.001). This increase was positive for all three sets of statements that were asked more than once (see Figure 2 [34,35]), indicating that participants became more accurate in their interpretation of the statements over time.

### Coronavirus-Related Behavior

Accurate beliefs were correlated with self-reported behavior aimed at preventing the coronavirus from spreading; *r* at all waves was between 0.26 and 0.29 and all *P* values were less than .001. This small but robust correlation suggests that accurate beliefs could be important for coronavirus-related behavior. We explored potential evidence of any causal effects in the data using a random-intercept cross-lagged panel model. This yielded a tentative indication that accurate beliefs might be predictive of behavior, with belief accuracy at T2 predicting coronavirus-related behavior at T3. However, with all other paths showing no sign of significant predictive effects, the results regarding causality are largely inconclusive (see Multimedia Appendix 5).

### Associations With Belief Accuracy

**Figure 3:**
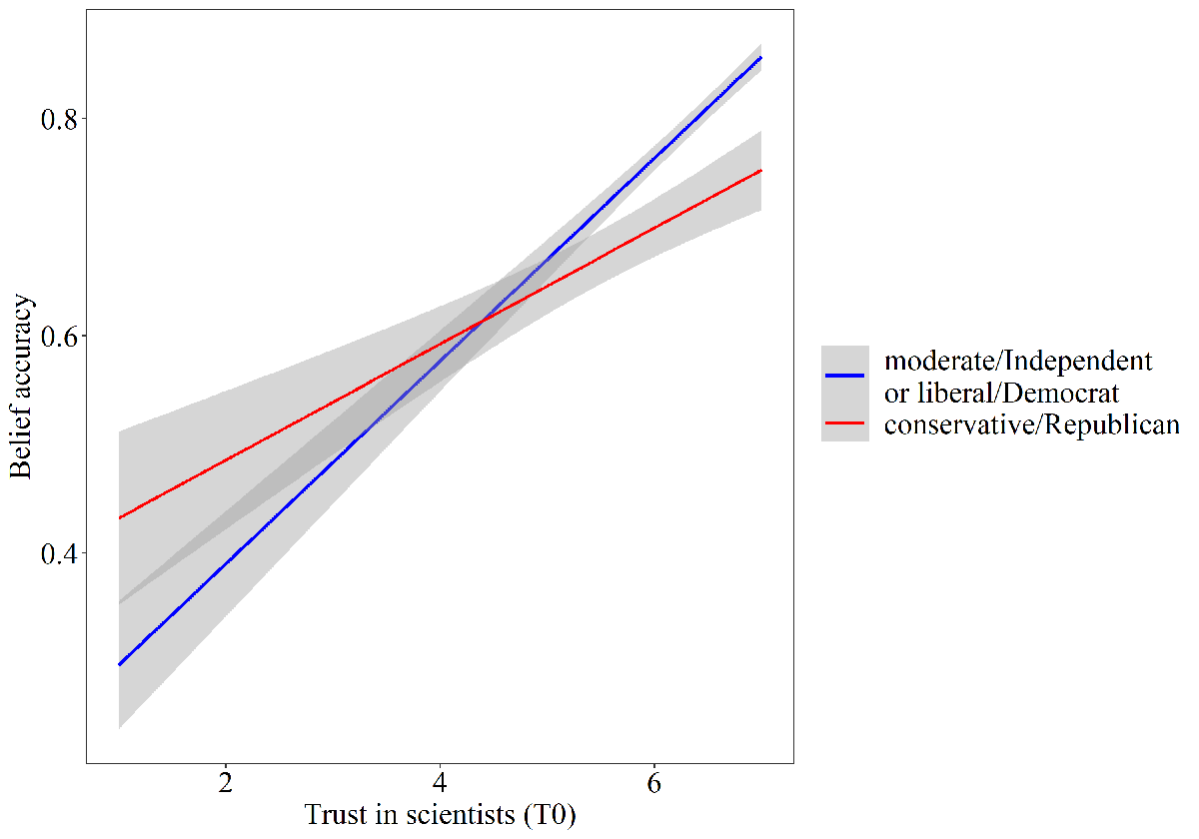
Linear relationship between belief accuracy (averaged over wave for plotting) and trust in scientists at T0 (baseline), split by political orientation (dichotomized for plotting). The grey area represents the 95% CI. The belief accuracy scale ranged from –1, indicating completely inaccurate beliefs, to 1, indicating completely accurate beliefs. Trust in scientists was measured with responses to the statement “I trust scientists as a source of information about the coronavirus.” Participants responded on a 7-point scale ranging from 1 (strongly disagree) to 7 (strongly agree).

We explored the relationship of trust in scientists at T0, political orientation, and the primary news source with belief accuracy. The mixed model yielded a significant positive relationship between belief accuracy and trust (estimate=0.07, SE<0.01; t_1200.23_=16.44, *P*<.001) and a significant negative correlation with political orientation (estimate=–0.02, SE<0.01; t_1199.62_=–6.78, *P*<.001). These main effects indicated that participants with higher trust in scientists scored higher on the measure of belief accuracy and that liberal, Democratic participants held more accurate beliefs than conservative, Republican participants. Moreover, these main effects were partially qualified by an interaction effect among trust and political orientation (estimate=–0.01, SE<0.01; t_1195.05_=–3.62, *P*<.001). Plotting of this interaction effect demonstrated that trust in scientists had a stronger relationship with belief accuracy for liberal, Democratic participants than it had for conservative, Republican participants (see Figure 3).

**Figure 4:**
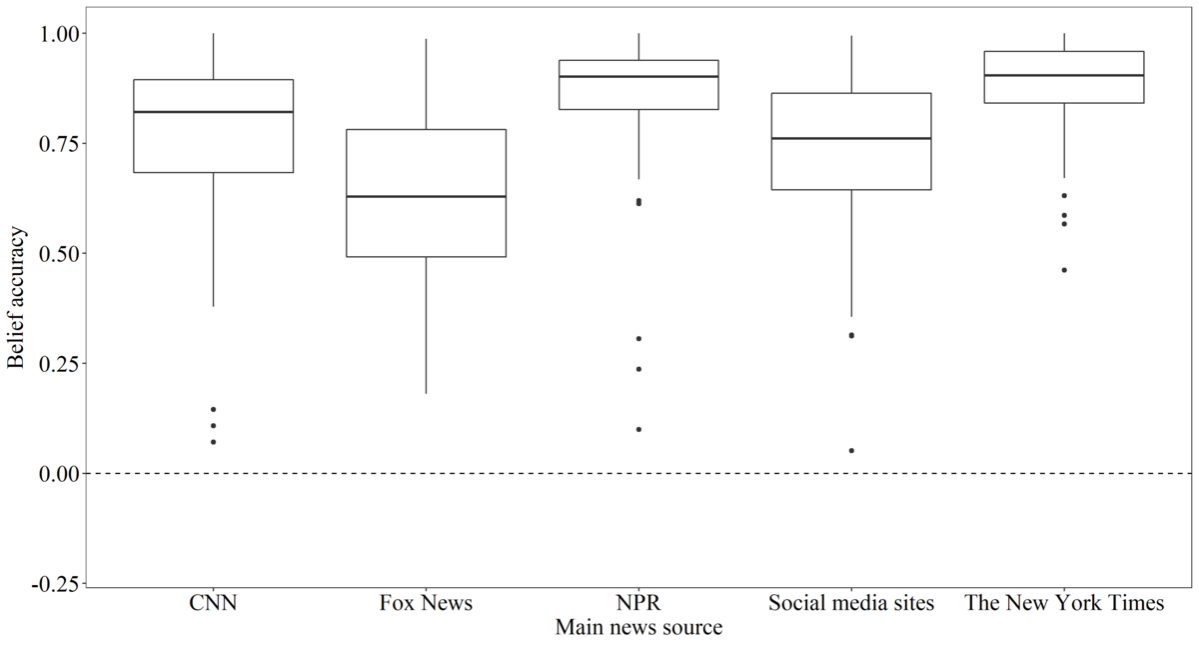
Box plot of raw (unadjusted) scores of belief accuracy (averaged over wave for plotting) by main news source. The belief accuracy scale ranged from –1, indicating completely inaccurate beliefs, to 1, indicating completely accurate beliefs.

Two of the five most chosen primary news sources were associated with a worse understanding of the facts regarding the COVID-19 pandemic than others (see Figure 4). Participants who reported CNN (estimate=–0.03, SE=0.01; t_1194.49_=–2.33, *P*=.02) or Fox News (estimate=–0.05, SE=0.02; t_1202.49_=–3.05, *P*=.002) as their main news source scored below average on belief accuracy.

### Intervention

We conducted a manipulation check and, as expected, when asked how they evaluated claims, participants in the boost condition (136/600, 22.7%) mentioned consensus, or something similar, more often than participants in the control condition (26/602, 4.3%; χ^2^_1_=85.2, *P*<.001, N=1202).

**Figure 5:**
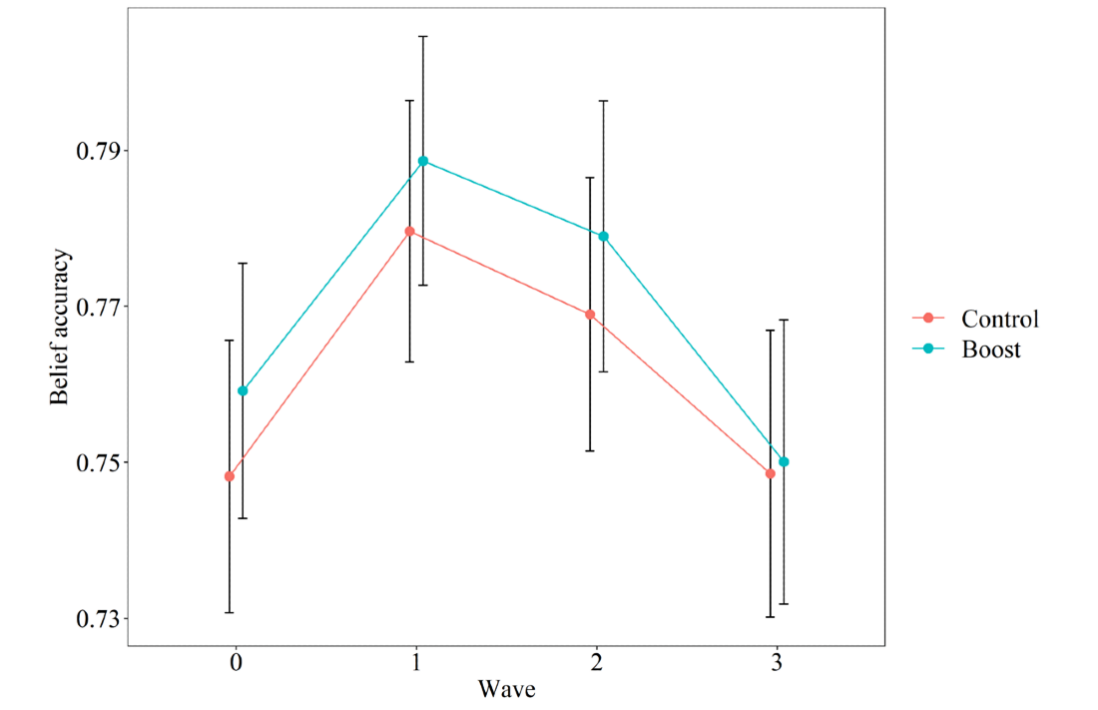
Belief accuracy per condition over time. Error bars indicate 95% CI focusing on the comparison between experimental conditions, not adjusted for within-subject variability. The belief accuracy scale ranged from –1, indicating completely inaccurate beliefs, to 1, indicating completely accurate beliefs.

We hypothesized that our boosting intervention would lead to more accurate beliefs about the COVID-19 pandemic than would the control condition. However, the interaction effect between condition and wave on belief accuracy was not significant (estimate<0.01, SE<0.01; t_1074.36_=0.22, *P*=.83). This means that the boosting intervention did not significantly alter belief accuracy of participants over time, compared to the control condition (see Figure 5). This was also the case when we explored effects of the intervention on inaccurate statements only (*P*=.48), accurate statements only (*P*=.49), only the original 10 statements that were included in all waves (*P*=.61), and only included participants who scored relatively low on belief accuracy at T0 (belief accuracy_T0_<0.76; *P*=.32).

When the subsample of participants in the boost condition who mentioned consensus, or similar, in the manipulation check was compared to participants in the control condition, we again found that the intervention did not increase belief accuracy (*P*=.21). However, there was a main effect of condition (estimate=0.06, SE=0.02; t_725.66_=3.50, *P*=.001), indicating that participants in the boost condition who did use consensus while evaluating claims scored higher on belief accuracy than participants in the control condition. This difference was already present at T0, so was not caused by the intervention.

**Figure 6:**
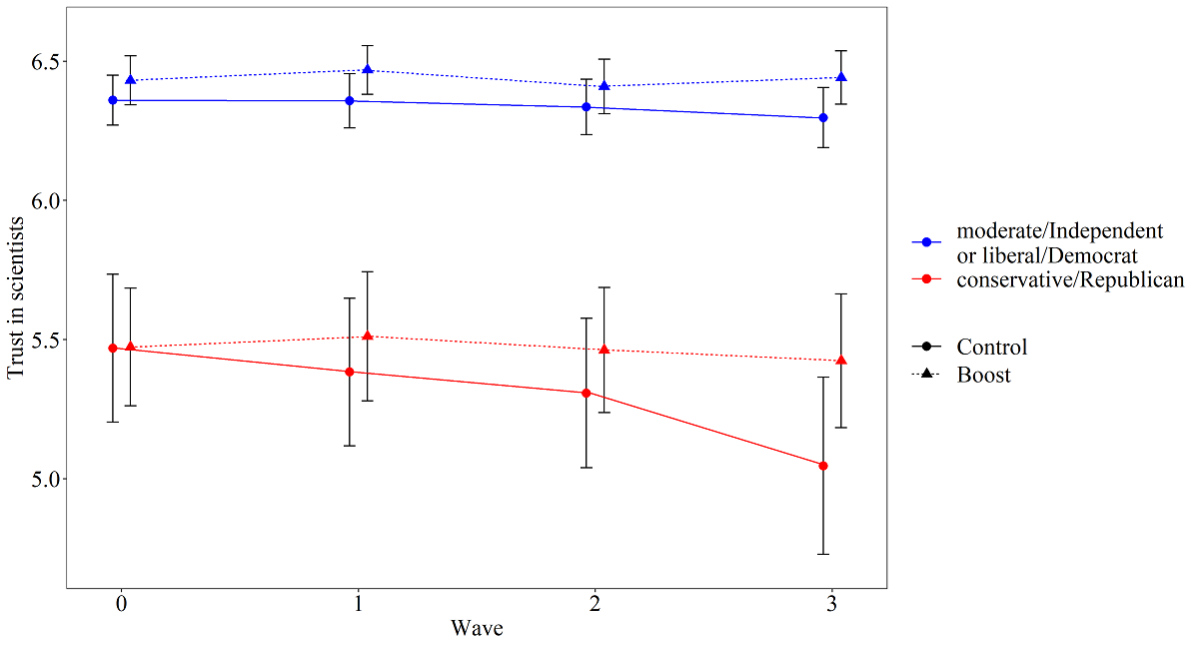
Trust in scientists as a source of information about the coronavirus per condition and political orientation (dichotomized for plotting) over time. Error bars indicate 95% CI focusing on the comparison between experimental conditions, not adjusted for within-subject variability. Trust in scientists was measured with responses to the statement “I trust scientists as a source of information about the coronavirus.” Participants responded on a 7-point scale ranging from 1 (strongly disagree) to 7 (strongly agree).

We explored the effect of the boosting intervention on trust in scientists as a source of information about the coronavirus. The mixed-effects model, similar to the hypothesis test but with the repeated measure of trust as the dependent variable and including the interaction term between condition, wave, and political orientation, yielded a significant three-way interaction effect between condition, wave, and political orientation (estimate=0.02, SE=0.01; t_1088.42_=2.39, *P*=.02). Trust in scientists was very high in all four waves (means between 6.11 and 6.19), but investigation of the two-way interaction effects per condition indicated a significant interaction effect among wave and political orientation in the control condition (estimate=–0.02, SE=0.01; t_562.01_=–3.24, *P*=.001), while there was no such significant interaction effect in the boost condition (*P*=.90). As illustrated by Figure 6, there was a clear overall difference in trust in scientists between participants related to their political orientation. More interestingly, trust remained stable for all participants in the boost condition, but decreased slightly for conservative, Republican participants in the control condition. This could indicate that the boosting intervention inhibited a decline of trust in scientists as a source of information about the coronavirus among more conservative, Republican participants.

## Discussion

### Principal Findings

The aims of this study were to gain insight into the beliefs of the US public about the COVID-19 pandemic and to investigate whether a boosting intervention could improve people’s belief accuracy. Interestingly, the average scores on belief accuracy over the surveyed 4-week period were high, indicating low belief in false statements and high belief in true statements. Looking at each set of statements, we found a small but significant increase in belief accuracy over time. This indicates that the general public is quite able to figure out what is true and what is not in times of crisis. Moreover, a small but robust correlation suggests that accurate beliefs about the pandemic might be important for coronavirus-related behavior. Associations with belief accuracy suggest that the processes of belief formation and correction might be affected by individuals’ trust in scientists and political orientation, as well as their news habits. Finally, the boosting intervention yielded no significant increase in belief accuracy over the control condition, demonstrating that the boosting infographic was not successful in helping people figure out what is true and what is false. Exploratory analyses suggested that the intervention did, however, inhibit a decline in trust in scientists as a source of information about the coronavirus among more conservative, Republican participants.

### Comparison With Prior Work

There is a great deal of worry about the prevalence of misinformation during the current pandemic, which is reflected in popular media (eg, [36,37]), as well as among scientists (eg, [38,39]) and public health agencies (eg, [4,40]). The supposed COVID-19 infodemic is not reflected in US citizens’ beliefs. The finding that most Americans hold quite accurate beliefs about the COVID-19 pandemic is in line with emerging work on perceptions of the pandemic that shows that belief in COVID-19 misperceptions and conspiracy theories is quite low [41-44]. Consequently, this calls into question the prevalence of misinformation or the public’s susceptibility to misinformation.

A convincing body of empirical work on the prevalence of misinformation surrounding the COVID-19 pandemic is not yet available. Research from before the COVID-19 pandemic indicates that the prevalence of misinformation might be lower than many believe [45-47]. Still, it is possible that the current pandemic has led to an increase in misinformation compared to the information landscape from before the pandemic. However, when looking for potential explanations of this study’s findings, we should consider the possibility that COVID-19 misinformation is not as prevalent as expected. Perhaps misinformation makes up only a small portion of the average US citizen’s media diet.

The second possibility is that we are indeed facing a COVID-19 infodemic, but that the public is not very susceptible to it. Misinformation campaigns regarding other topics, such as climate change and the health effects of tobacco [18,48], have demonstrated that misinformation can contribute to misperceptions about important matters. In these cases, however, misinformation campaigns have been carefully organized and executed, continually misinforming the public for decades. In contrast, the COVID-19 pandemic is a novel issue and, at least in the relatively early months that we investigated, did not yield many such coordinated misinformation campaigns. Moreover, the COVID-19 pandemic originated in a very different media landscape than the climate change and tobacco misinformation campaigns. Fake news, misinformation, and disinformation have been discussed widely and frequently in popular media since the 2016 US presidential election and the 2016 United Kingdom European Union membership referendum. This might have resulted in the public being more aware of misinformation campaigns targeting them. Perhaps the widespread discussion of misinformation in popular media has worked as a large-scale media literacy intervention, putting people *on guard* against false information. In support of this idea, recent research has demonstrated that simply asking one to consider the accuracy of a claim improved subsequent choices about what COVID-19 news to share on social media [49].

A third possibility that should be considered is that the public is more careful in forming beliefs in times of crisis, especially in the relatively early days of a crisis, making a well-informed public not unique to the COVID-19 pandemic. In times of crisis, people are likely to increase news consumption (eg, [50]). This was also the case in the United States during the first months of the COVID-19 pandemic, with people reporting increased news consumption [51]. Although we found that those who reported CNN or Fox News as their main news source scored below average on belief accuracy, the general increase in news consumption may lead to a better understanding of the crisis situation, including more accurate beliefs.

Turning to the finding that political orientation is associated with individuals’ belief accuracy, we see that this is in line with other emerging work [41]. There is likely a multitude of explanations for this evolving partisan divide [52] regarding perceptions about the pandemic, such as political party cues in the news affecting opinion formation [53], the difficulty of correcting false beliefs for the ideological group most likely to hold those misperceptions [17], as well as the differences in news consumption that are reflected in this study. A second variable that is even more strongly related to belief accuracy is trust in scientists as a source of information about the coronavirus, demonstrating that higher trust is related to more accurate beliefs. Interestingly, the associations of political orientation and trust with accurate beliefs were partially explained by an interaction effect among political orientation and trust. The stronger association of trust with belief accuracy for more liberal, Democratic individuals might mean that they rely more on scientists’ perceptions in forming beliefs, while relatively more conservative, Republican individuals might rely more on other cues. Relatedly, the inhibited decline in trust in scientists among conservative, Republican participants in the boost condition could indicate that information about the scientific process might resonate more with them than just hearing the results of this process. Though this exploratory finding should be replicated, it could provide a fruitful avenue for further research on trust in scientists and political orientation.

In addition, this study demonstrates that some news sources might be doing a worse job of informing their consumers about the COVID-19 pandemic than others, or perhaps that better-informed news consumers turn to different news sources than less well-informed consumers, again in line with other emerging work [41]. Most likely, a combination of both selection and influence (eg, [54]) explain the differences in belief accuracy found in this study. Interestingly, considering the role of social media in the spread of misinformation (eg, [55]), with about 26% to 42% of tweets in the data collection period containing unreliable facts [56], participants who reported social media sites as their main source of news about the coronavirus did not display significantly worse belief accuracy than others. However, it is possible that participants who reported social media sites as their main source followed major news outlets via the social media site, thereby being exposed to similar news content as the other participants.

Finally, this study demonstrates the difficulty of crafting interventions aimed at increasing belief accuracy. Recent work demonstrates that simple, short media literacy interventions can work [14,15], while other work highlights the difficulties of crafting these interventions [16]. We argue that the divergent findings can be explained by the fact that in the former work the interventions were paired with corrections, while in this study participants had to put their new skill to use outside of the study context. Considering that cues signaling the existence of consensus in relevant news content are very rare [57], participants likely had to search for information about scientific consensus themselves. The results from the manipulation check indicated that only a relatively small portion of participants actually applied this strategy. However, those individuals who indicated that they did apply a strategy related to consensus reasoning scored higher on belief accuracy than the control group. This difference highlights the potential of the intervention in situations where individuals can be empowered to actually apply it.

### Limitations

There are two notable limitations to this study. First, our belief accuracy measure consisted only of science-based statements. We incorporated only science-based claims in our study to ensure that there was sufficient empirical evidence stating that a claim was either true or false. However, this decision did exclude some coronavirus-related claims that were not based on science (eg, “Bill Gates patented the coronavirus”) or were unresolved at the time (eg, “A vaccine will be available before the end of the year”). It should have been harder for participants to figure out whether such unresolved issues were true or not, yielding different responses from participants for a measure reflecting non-science-based, unresolved issues about the pandemic.

A second limitation is the fact that the recruitment platform that we used, Prolific, is known as a platform for research. Although participants on the platform receive financial incentives for completing studies, they might be more interested in scientific research than the average US citizen. This could lead to them also having a higher trust in science than the general population, even though our sample was balanced regarding age, gender, and ethnicity. As trust in science was highly related to belief accuracy, it could be possible that this led to an inflated belief accuracy score. Future research should attempt to replicate this study with a sample that represents the US population better than our balanced sample.

### Conclusions

Our work demonstrates that most people are quite able to figure out the facts in this time of crisis, but also that it is difficult to craft an intervention targeting these beliefs. However, in cases where people do not immediately have a clear understanding of the facts, they are capable of figuring them out over time. There are some factors that might make it easier or harder for one to figure out the facts. We found that the accuracy of participants’ beliefs was related to political orientation, as well as their primary news source. This suggests that, even in the relatively early days of the pandemic, political polarization and media diet had a grip on US citizens’ factual beliefs, leading to polarization along party lines. Another factor strongly related to accurate beliefs about the pandemic was trust in scientists. It is unclear whether an already-high trust led to accurate beliefs or whether being able to figure out the facts increased trust in scientists, but the importance of expert communication is underlined by these findings.

Although a small but robust correlation suggests that accurate beliefs about the pandemic might be important for coronavirus-related behavior, the role of misinformation in the pandemic seems to be relatively small, either because it is rare or because it is unable to persuade. However, we note that even if misinformation is not prevalent and only accepted by a small portion of the receivers, it can still be dangerous. To illustrate, we found that almost all participants in this study disregarded the statement that injecting or ingesting bleach is a safe way to kill the coronavirus, but this false claim is reported to have cost at least one life [58]. Additionally, with the antivaccine community launching coordinated misinformation campaigns against potential coronavirus vaccines [59] and politicization of the pandemic looming, the infodemic might become a much bigger threat.

## Appendix

Multimedia Appendix 1 Attention and seriousness checks.

Multimedia Appendix 2 Resources used to collect COVID-19 pandemic belief statements.

Multimedia Appendix 3 Infographic used in the boosting intervention to empower participants to use the scientific consensus when evaluating claims related to the COVID-19 pandemic.

Multimedia Appendix 4 Descriptive statistics of all statements per wave.

Multimedia Appendix 5 Random-intercept cross-lagged panel model (RI-CLPM).

## Abbreviations

OSF: Open Science Framework
T0: baseline
T1: first follow-up wave
T2: second follow-up wave
T3: final wave
WHO: World Health Organization
