# Supplementary material for: Investigating and Improving the Accuracy of US Citizens’ Beliefs About the COVID-19 Pandemic: Longitudinal Survey Study
Source: J Med Internet Res. 2021 Jan 12;23(1):e24069. doi: 10.2196/24069 (PMC7806340; doi:10.2196/24069)
Supplement: Multimedia Appendix 1 [file jmir_v23i1e24069_app1.docx]

## Multimedia Appendix 1: Attention and seriousness checks.

An attention check was included in the measure of belief accuracy in all waves. Attention checks are used to filter out careless responding, which has been demonstrated to improve the quality of survey data [1]. The attention check consisted of an instructed-response item, which stated “To demonstrate that you are paying attention, please answer "False". Furthermore, at T0, a seriousness check was included. Seriousness checks are also used to improve data quality [2]. The seriousness check consisted of telling participants that as researchers, the quality of their data was very important to us, so we wanted to make sure that their responses were valid and authentic. We asked them “In your honest opinion, should we use your data?” Participants responded with either “Yes” or “No”.

## References

1. Meade AW, Craig SB. Identifying careless responses in survey data. Psychol Methods 2012;17(3):437–455. PMID:22506584

2. Aust F, Diedenhofen B, Ullrich S, Musch J. Seriousness checks are useful to improve data validity in online research. Behav Res Methods 2013;45:527–535. [doi: https://doi.org/10.3758/s13428-012-0265-2]
