# Supplementary material for: Investigating and Improving the Accuracy of US Citizens’ Beliefs About the COVID-19 Pandemic: Longitudinal Survey Study
Source: J Med Internet Res. 2021 Jan 12;23(1):e24069. doi: 10.2196/24069 (PMC7806340; doi:10.2196/24069)
Supplement: Multimedia Appendix 2 [file jmir_v23i1e24069_app2.docx]

## Multimedia Appendix 2: Resources used to collect COVID-19 pandemic belief statements.

Empirical research:

- Preprint by Pennycook et al.: [1]
- Preprint by Singh et al.: [2]

Media tracking organizations:

- RCAID COVID-19 Insights Center: https://covid19.rcaid.org/
- NewsGuard: https://www.newsguardtech.com/covid-19-myths/

Expert reports in established media:

- BBC Reality Check: https://www.bbc.com/news/reality_check
- CNBC Expert Debunking: https://www.cnbc.com/2020/05/01/experts-explain-why-coronavirus-myths-misinformation-can-be-dangerous.html
- The Guardian: What do scientists know: https://www.theguardian.com/world/2020/apr/30/coronavirus-what-do-scientists-know-about-covid-19-so-far
- The Guardian: Coronavirus myths busted: https://www.theguardian.com/world/2020/apr/11/can-a-face-mask-protect-me-from-coronavirus-covid-19-myths-busted

Public health agencies and medical institutes:

- WHO myth busters: https://www.who.int/emergencies/diseases/novel-coronavirus-2019/advice-for-public/myth-busters
- Harvard Medical School Coronavirus Resource Center: https://www.health.harvard.edu/diseases-and-conditions/coronavirus-resource-center

## References

1. Pennycook G, McPhetres J, Bago B, Rand DG. Attitudes about COVID-19 in Canada, the U.K., and the U.S.A.: A novel test of political polarization and motivated reasoning [Internet]. 2020. [doi: 10.31234/osf.io/zhjkp]

2. Singh L, Bansal S, Bode L, Budak C, Chi G, Kawintiranon K, Padden C, Vanarsdall R, Vraga E, Wang Y. A first look at COVID-19 information and misinformation sharing on Twitter. Preprint [Internet] 2020 Mar 30; PMID:32550244
