# Supplementary figures and images for: Investigating and Improving the Accuracy of US Citizens’ Beliefs About the COVID-19 Pandemic: Longitudinal Survey Study
Source: J Med Internet Res. 2021 Jan 12;23(1):e24069. doi: 10.2196/24069 (PMC7806340; doi:10.2196/24069)

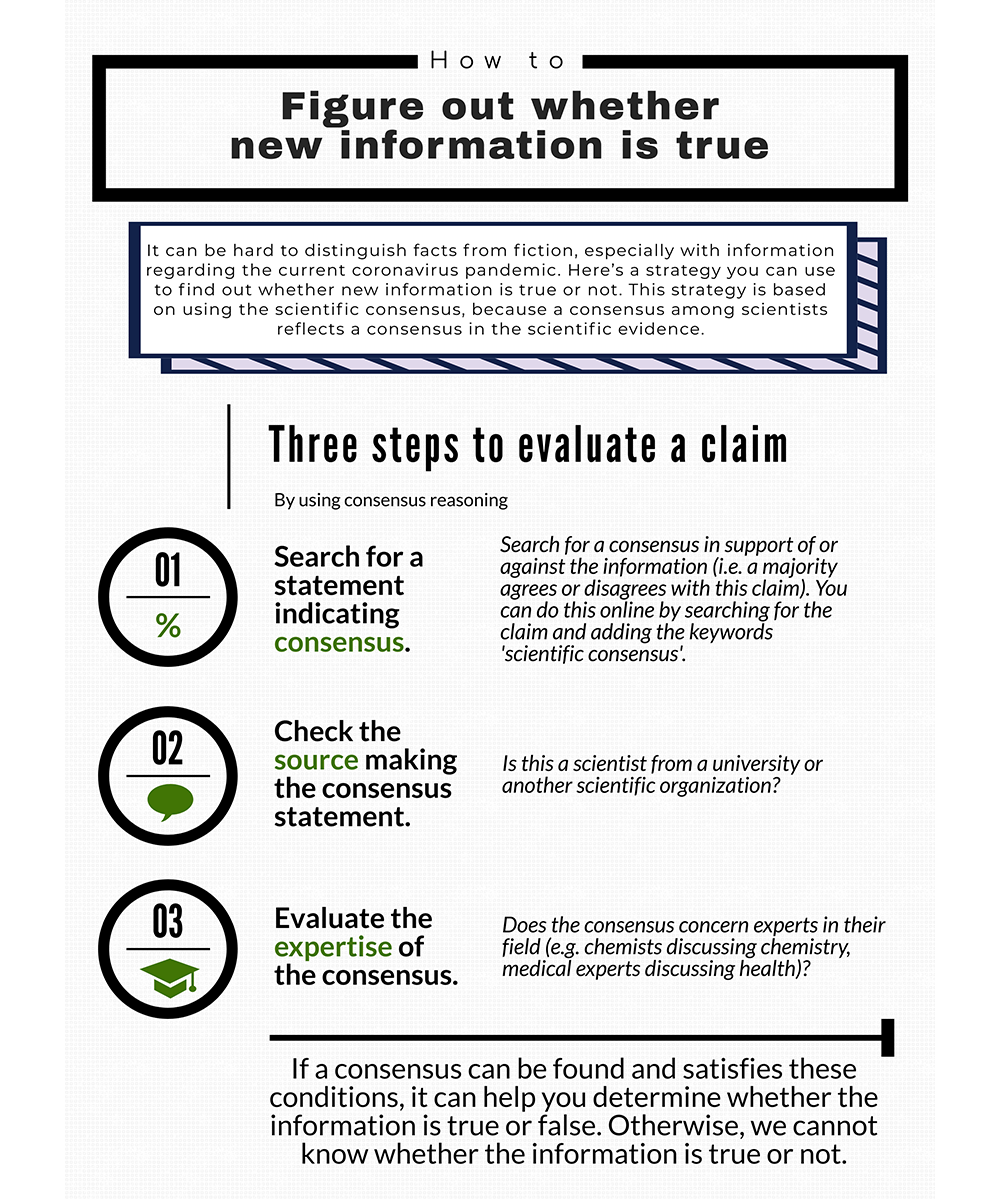

Supplement: Multimedia Appendix 3 [file jmir_v23i1e24069_app3.png]
