# Supplementary material for: Investigating and Improving the Accuracy of US Citizens’ Beliefs About the COVID-19 Pandemic: Longitudinal Survey Study
Source: J Med Internet Res. 2021 Jan 12;23(1):e24069. doi: 10.2196/24069 (PMC7806340; doi:10.2196/24069)
Supplement: Multimedia Appendix 4 [file jmir_v23i1e24069_app4.docx]

## Multimedia Appendix 4: Descriptive statistics of all statements per wave.

Table S1. Mean belief accuracy (and SD) per belief statement per wave.

| Statement | Wave | | | |
| --- | --- | --- | --- | --- |
|  | Wave 0 (N=1202) | Wave 1 (n=1078) | Wave 2 (n=1067) | Wave 3 (n=1022) |
| Radiation from 5G cell towers is helping spread the coronavirus | 0.85 (0.35) | 0.86 (0.34) | 0.86 (0.33) | 0.87 (0.33) |
| The coronavirus is man-made | 0.52 (0.55) | 0.52 (0.55) | 0.56 (0.54) | 0.57 (0.54) |
| Complementary products, such as colloidal silver or herbal remedies, have been proven effective in preventing or treating COVID-19 | 0.73 (0.44) | 0.74 (0.44) | 0.76 (0.44) | 0.77 (0.43) |
| The coronavirus is a biological weapon developed by the Chinese government | 0.60 (0.51) | 0.62 (0.50) | 0.64 (0.49) | 0.64 (0.50) |
| A safe and effective vaccine for COVID-19 is available at this time | 0.90 (0.32) | 0.89 (0.32) | 0.89 (0.31) | 0.88 (0.34) |
| The name given to the novel 2019 coronavirus is Severe Acute Respiratory Syndrome Coronavirus-2 (SARS-CoV-2) | 0.28 (0.72) | 0.42 (0.72) | 0.54 (0.65) | 0.62 (0.62) |
| Regular hand washing can help prevent the spread of the coronavirus | 0.92 (0.22) | 0.93 (0.21) | 0.92 (0.22) | 0.93 (0.23) |
| Fever is one of the symptoms of COVID-19 | 0.91 (0.25) | 0.91 (0.30) | 0.92 (0.25) | 0.92 (0.26) |
| The elderly are at a higher risk of becoming severely ill due to COVID-19 | 0.92 (0.24) | 0.93 (0.23) | 0.93 (0.25) | 0.93 (0.24) |
| Social distancing helps slow the spread of the coronavirus | 0.91 (0.26) | 0.91 (0.26) | 0.91 (0.26) | 0.90 (0.28) |
| Injecting or digesting bleach is a safe way to kill the coronavirus | – | 0.97 (0.19) | 0.96 (0.21) | 0.97 (0.19) |
| Warm weather stops the coronavirus from spreading entirely | – | 0.61 (0.51) | 0.62 (0.51) | 0.64 (0.51) |
| The coronavirus spreads mainly from person-to-person | – | 0.83 (0.33) | 0.84 (0.32) | 0.88 (0.28) |
| There is a delay between the moment a person is first infected with the coronavirus and the time this person develops symptoms | – | 0.84 (0.34) | 0.87 (0.30) | 0.87 (0.32) |
| The vast majority of people who contract the coronavirus will need to be hospitalized | – | – | 0.58 (0.58) | 0.60 (0.58) |
| There is overwhelming evidence for the safety and effectiveness of hydroxychloroquine in treating COVID-19 | – | – | 0.58 (0.57) | 0.63 (0.56) |
| Some people who have been infected with the coronavirus have no symptoms | – | – | 0.88 (0.34) | 0.90 (0.30) |
| COVID-19 is more deadly than the seasonal flu | – | – | 0.66 (0.54) | 0.67 (0.54) |
| Pets are a source of infection with the coronavirus by spreading the virus to humans | – | – | – | 0.51 (0.55) |
| There is strong evidence that vitamin C can cure COVID-19 | – | – | – | 0.76 (0.42) |
| The coronavirus originated in wildlife | – | – | – | 0.32 (0.60) |
| Currently there is no specific effective antiviral treatment for COVID-19 | – | – | – | 0.70 (0.51) |
