# Supplementary material for: Investigating and Improving the Accuracy of US Citizens’ Beliefs About the COVID-19 Pandemic: Longitudinal Survey Study
Source: J Med Internet Res. 2021 Jan 12;23(1):e24069. doi: 10.2196/24069 (PMC7806340; doi:10.2196/24069)
Supplement: Multimedia Appendix 5 [file jmir_v23i1e24069_app5.docx]

## Multimedia Appendix 5: Random intercept cross-lagged panel model (RI-CLPM).

We used a RI-CLPM to explore the relationship between the accuracy of participants’ coronavirus and COVID-19 beliefs and their reported coronavirus-related behavior. RI-CLPMs are an analytical strategy used to describe directional influences between variables over time, focusing on the within-person variation [1]. We fit a model in which the means of each variable were unconstrained over time, including the variance and covariance over time. The fit measures indicated good model fit (RMSEA=0.021, SRMR=0.012, CFI=0.999). Only one of the cross-lagged paths was statistically significant, indicating that belief accuracy at T2 might be predictive of coronavirus-related behavior at T3 (see Table S1). Note that the autoregressive path of coronavirus-related behavior at T2 was still significant as well.

Table S1. Regression paths in RI-CLPM exploring the relationship between belief accuracy and coronavirus-related behavior.^a^

| Outcome | Predictors | Estimate | SE | *Z* | *P* |
| --- | --- | --- | --- | --- | --- |
|  |  |  |  |  |  |
| **BA T3** |  |  |  |  |  |
|  | BA T2 | 0.50 | 0.07 | 6.94 | <.001 |
|  | CB T2 | -0.01 | 0.02 | -0.63 | .53 |
| **BA T2** |  |  |  |  |  |
|  | BA T1 | 0.04 | 0.15 | 0.26 | .79 |
|  | CB T1 | 0.03 | 0.03 | 1.04 | .30 |
| **BA T1** |  |  |  |  |  |
|  | BA T0 | 0.00 | 0.07 | 0.04 | .96 |
|  | CB T0 | -0.03 | 0.03 | -1.00 | .32 |
| **CB T3** |  |  |  |  |  |
|  | CB T2 | 0.39 | 0.09 | 4.16 | <.001 |
|  | BA T2 | 0.74 | 0.30 | 2.44 | .015 |
| **CB T2** |  |  |  |  |  |
|  | CB T1 | 0.13 | 0.13 | 0.97 | .33 |
|  | BA T1 | -0.14 | 0.56 | -0.26 | .80 |
| **CB T1** |  |  |  |  |  |
|  | CB T0 | -0.23 | 0.22 | -1.05 | .29 |
|  | BA T0 | 0.02 | 0.25 | 0.10 | .92 |

^a^BA = belief accuracy, CB = coronavirus-related behavior.

## References

1. Hamaker EL, Kuiper RM, Grasman RPPP. A critique of the cross-lagged panel model. Psychol Methods [Internet] 2015;20(1):102–116. [doi: 10.1037/a0038889]
